# Interstitial lung disease in patients with non-small-cell lung cancer: causes, mechanisms and management

**DOI:** 10.1038/sj.bjc.6602060

**Published:** 2004-08-31

**Authors:** P Camus

**Affiliations:** 1 Services de Pneumologie, University Hospital and Medical School, Université de Bourgogne, Dijon, France

The incidence of lung cancer has markedly increased in the past few decades and is still increasing in many countries worldwide. Lung cancer is a leading cause of death in many developed countries, and approximately 140 000 new cases are identified each year in the US alone (Green, 2004). The majority of patients with non-small-cell lung cancer (NSCLC) are diagnosed with advanced disease and cannot undergo radical surgical treatment. Typically, patients from this large group are offered best supportive care or palliative treatment regimens incorporating various combinations of chemotherapy and radiotherapy.

Progress in palliative care for lung cancer has been slow; however, advances have been made due to better utilisation of drugs (i.e. timing, duration of treatment), choice of drugs for first-line therapy and subsequent regimens, and optimal combination and timing with radiation therapy (Spiro and Porter, 2002). Chemotherapy or chemoradiation therapy is the treatment of choice for unresected lung cancer in many countries (Spiro and Porter, 2002) and may be accepted as a treatment option for resected lung cancer in the future (Arriagada *et al*, 2004). There is the general agreement that properly used chemotherapy regimens have increased life expectancy and improved the quality of life of patients with lung cancer.

However, chemotherapy benefits can be at the expense of adverse effects in different organ systems, including the lung (Foucher *et al*, 2003; Limper, 2004). Several novel compounds, such as irinotecan (Masuda *et al*, 1992; Foucher *et al*, 2003), gemcitabine (Pavlakis *et al*, 1997), paclitaxel (Goldberg and Vannice, 1995) and docetaxel (Merad *et al*, 1997), have been associated with the development of sometimes severe lung disease in about 3–5% of patients with lung cancer (Kunitoh *et al*, 1996; Merad *et al*, 1997; Wang *et al*, 2001; Erasmus *et al*, 2002; Lilly, 2003). The assessment of drug causality in patients with lung cancer who develop interstitial lung disease (ILD) is difficult compared with other respiratory reactions to drugs. The diagnosis of drug-associated lung disease is against the background of the underlying neoplastic lung disease, adverse effects of other drugs, colony-stimulating factors, oxygen or radiation therapy and opportunistic infections. Patients with lung cancer are often sequentially exposed to different chemotherapy regimens and the risk of drug-induced disease may increase correlatively. Clarifying the respective role of these factors is often impossible.

Gefitinib (‘Iressa’) is a new type of targeted treatment for NSCLC. It is an inhibitor of the epidermal growth factor receptor (EGFR) signalling pathway that acts intracellularly at the level of the EGFR tyrosine kinase. Two phase II monotherapy trials (‘Iressa’ Dose Evaluation in Advanced Lung cancer [IDEAL] 1 and 2) have reported unprecedented antitumour activity and symptom relief in pretreated patients with advanced metastatic NSCLC (Fukuoka *et al*, 2003; Kris *et al*, 2003). Furthermore, recent evidence in patients with NSCLC points to a specific gain in function in patients who bear somatic mutations in the tyrosine kinase domain of the EGFR (Lynch *et al*, 2004; Paez *et al*, 2004). These mutations exert a dominant oncogenic effect that correlates with the clinical response to gefitinib, contrasted with the low response rate seen in patients lacking these mutations (Lynch *et al*, 2004; Paez *et al*, 2004). However, in addition to patients with a clinical response, an additional 35% of patients experience stable disease or symptom improvement with gefitinib (Fukuoka *et al*, 2003; Kris *et al*, 2003) and further molecular studies are clearly required to identify predictive markers in this group of patients. The prevalence of this mutation is also different in Japanese NSCLC patients, as opposed to NSCLC patients from the US (Paez *et al*, 2004), which may explain the greater response rate of Japanese patients to gefitinib.

Although gefitinib is not associated with many of the general adverse effects of broadly acting cytotoxic chemotherapeutic agents, recent reports from Japan have indicated that a proportion of patients treated with gefitinib experienced severe ILD (Ieki *et al*, 2003; Inoue *et al*, 2003; Okamoto and Suga, 2003; Rabinowits *et al*, 2003; Sumter *et al*, 2003). Evaluation of drug causality is also difficult here. Tolerability data from the compassionate use of gefitinib in the ‘Iressa’ Expanded Access Programme support the favourable safety profile reported in phase I and II trials, and incidence figures for ILD in the West are not dissimilar to those reported for gefitinib- *vs* placebo-exposed patients with NSCLC (Giaccone *et al*, 2002; Herbst *et al*, 2004). Whether the mutations of the EGFR signalling domain referred to above also modulate the occurrence of adverse pulmonary effects of gefitinib, or interfere with repair processes of the pulmonary epithelium (Suzuki *et al*, 2003), is unknown at this time.

This experience of ILD in patients with NSCLC has posed a number of important questions relating to definition, diagnosis, management and mechanisms. A group of experts in the field of NSCLC and lung disease discussed these issues at a symposium held in Seattle in May 2003. The content of the presentations is discussed within this supplement. Hopefully, the knowledge of gefitinib may provide new insights into the field of ILD and chemotherapy-associated lung disease in the way it has in the treatment of cancer.
